# A pilot randomized controlled trial of EKG for neonatal resuscitation

**DOI:** 10.1371/journal.pone.0187730

**Published:** 2017-11-03

**Authors:** Anup Katheria, Kathy Arnell, Melissa Brown, Kasim Hassen, Mauricio Maldonado, Wade Rich, Neil Finer

**Affiliations:** Neonatal Research Institute, Sharp Mary Birch Hospital, San Diego, CA, United States of America; TNO, NETHERLANDS

## Abstract

**Background:**

The seventh edition of the American Academy of Pediatrics Neonatal Resuscitation Program recommends the use of a cardiac monitor in infants that need resuscitation. Previous trials have shown that EKG heart rate is available before pulse rate from a pulse oximeter. To date no trial has looked at how the availability of electrocardiogram (EKG) affects clinical interventions in the delivery room.

**Objective:**

To determine whether the availability of an EKG heart rate value and tracing to the clinical team has an effect on physiologic measures and related interventions during the stabilization of preterm infants.

**Design/Methods:**

Forty (40) premature infants enrolled in a neuro-monitoring study (The Neu-Prem Trial: NCT02605733) who had an EKG monitor available were randomized to have the heart rate information from the bedside EKG monitor either displayed or not displayed to the clinical team. Heart rate, oxygen saturation, FiO_2_ and mean airway pressure from a data acquisition system were recorded every 2 seconds. Results were averaged over 30 seconds and the differences analyzed using two-tailed t-test. Interventions analyzed included time to first change in FiO_2_, first positive pressure ventilation, first increase in airway pressure, and first intubation.

**Results:**

There were no significant differences in time to clinical interventions between the blinded and unblinded group, despite the unblinded group having access to a visible heart rate at 66 +/- 20 compared to 114 +/- 39 seconds for the blinded group (p < .0001). Pulse rate from oximeter was lower than EKG heart rate during the first 2 minutes of life, but this was not significant.

**Conclusion(s):**

EKG provides an earlier, and more accurate heart rate than pulse rate from an oximeter during stabilization of preterm infants, allowing earlier intervention. All interventions were started earlier in the unblinded EKG group but these numbers were not significant in this small trial. Earlier EKG placement before pulse oximeter placement may affect other interventions, but this needs further study.

## Introduction

The majority of newborn infants make the transition from the intrauterine to extrauterine environment successfully; however, approximately 10% of newborn infants require assistance during this transition. Current guidelines from the American Academy of Pediatrics (AAP) and the International Liaison Committee of Resuscitation (ILCOR) state the importance of heart rate (HR) as the most vital of vital signs during neonatal transition and/or resuscitation. In infants who are severely depressed at birth, continually updated heart rate provides caregivers the opportunity to respond to HR as it happens, rather than learning it the next time someone auscultates or palpates the heart rate.[1] Several studies have demonstrated that HR by auscultation or palpation of the umbilical cord are inaccurate.[2–4] HR measurements by pulse oximetry are superior to auscultation and palpation, but there is a period where the HR may be inaccurate until an adequate wave form is achieved.[5] HR from a hand held Doppler has been shown to correlate reasonably well with EKG and pulse oximetry (PO) heart rates, [2, 6] however this is not practical for continuous HR readings. Several non-contact methods are currently under investigation but are not readily available as a tool to collect continuous HR data, including photoplethysmography and non-contact Doppler radar.[7, 8] Electrocardiogram (EKG) provides a reliable HR faster than pulse oximetry.[5, 9, 10] In addition, one study has shown that pulse oximetry in the first 2 minutes of life frequently displayed the newborn’s HR below 60 beats per minute or 100 beats per minute, while a simultaneous EKG showed the HR greater than 100 beats per minute.[11] The recent guidelines from the International Liaison Committee on Resuscitation recommend the use of a 3-lead EKG for rapid and accurate assessment of a newborn’s HR. While the availability of an earlier, more accurate HR would seem useful on the surface, it has not been established whether this information has an impact on the timing or initiation of different resuscitation interventions. Therefore, we conducted a randomized trial that displayed or did not display EKG values to the clinical team to determine whether it had an effect on resuscitation interventions and physiologic parameters. Our primary outcome was to detect a difference in the time for various resuscitation interventions which included the time to increase supplemental oxygen, initiate positive pressure ventilation, or to perform endotracheal intubation.

## Methods

**Fig 1:**
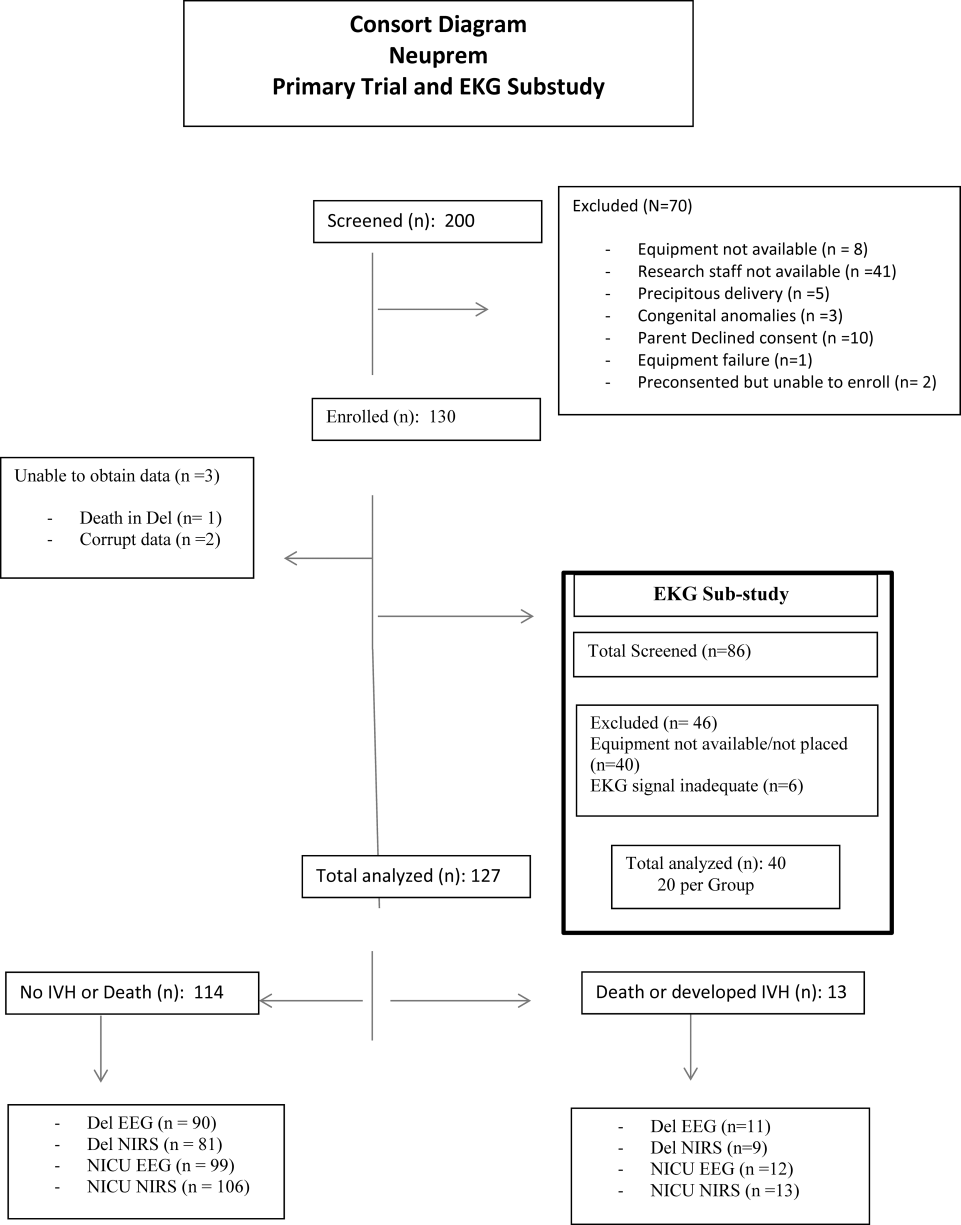
Consort diagram.

Subjects enrolled in this trial were part of a larger observational delivery room study (Neuromonitoring of Preterm Newborn Brain During Birth Resuscitation—The Neu-PremTrial: NCT02605733). Neu-Prem was an observational study to determine whether electroencephalogram (EEG) and near-infrared spectrocopy NIRS monitoring at birth could predict which infants would develop intraventricular hemorrhage and death. 127 infants were enrolled in Neu-Prem at Sharp Mary Birch Hospital for Women and Newborns. A consort diagram including both the primary trial and this substudy (Fig 1) is attached below, Supplementary documents including the data spreadsheet (S1 File), Consort questionnaire (S2 File) and Neuprem protocol with substudy (S3 File) are attached as supplementary information files (. The last 86 infants enrolled in the Neuprem trial were randomized to participate in the EKG sub-study. Of the 86 randomized, 40 had adequate EKG signals to be included.

The primary Neu-Prem trial and this EKG sub-study were approved by the Sharp Mary Birch Hospital for Women and Newborns Institutional Review Board, and written informed consent, which included participation in both studies, was obtained from the parents or guardians of participants prior to delivery when possible. If it was not possible to obtain antenatal consent, the IRB approved deferred consent whereby parents were approached after delivery to determine if they would approve of the data from their infant being used in the trial.

Infants were included in the sub-study if EKG leads were placed at the time of delivery. Infants in the EKG sub-study were randomized to a Non-displayed or Displayed group using opaque envelopes in the Delivery Room by a member of the research team if present, or by an Advanced Life Support nurse if no research team member was present. The Non-displayed group used standard methods (auscultation and/or pulse oximeter (PO)) to determine HR without the addition of a visible HR from the EKG. The Displayed group had the EKG monitor screen available to them during the entire resuscitation.

We developed a real-time data acquisition system, which includes video and analog data for all monitored parameters. The infant’s pulse rate and oxygen saturation were collected from the pulse oximeter (Radical-7, Masimo Corporation, Irvine, CA). EKG readings were obtained from the defibrillator output of the HR monitor (GE Dash 5000, GE Healthcare, Aurora, OH). Data were converted from analog to digital using a universal interface module attached to an MP150 data acquisition system (Biopac Systems, Inc, Goleta, CA). All channels were sampled at 200 samples per second (Hz), and the files were linked to the video to allow for review of temporal relationships. The camera was a Logitech C920 HD Pro webcam recording at a resolution of 1080p. Infant data were collected from the time the infant was placed on the warmer until either the infant was removed from the bed to be taken to the nursery or the face of the pulse oximeter was removed. For purposes of this study, we analyzed the portion of these data from the time the infant was placed on the bed until both monitoring devices (PO & EKG) were providing audible indicators of HR. The time to place EKG leads was calculated from the time the infant was placed on the bed until the third EKG lead was placed. The time to place the pulse oximeter was calculated from the time the infant was placed on the bed until the pulse oximeter sensor was connected.

## Lead/Electrode placement

Before delivery, the electrographic lead wires (Red Dot, 3M, St Paul, MN) were removed from the packet by a neonatal nurse and connected to the HR monitor. When the infant was placed on the radiant warmer after delivery, the infant’s chest was briefly dried, and 1 EKG lead was placed on the right side of the chest and 2 EKG leads were placed on the left side of the infant, one on the chest and one on the abdomen. Infants < 28 weeks’ gestation were placed in a polyethylene wrap immediately after drying. The plastic wrap was briefly pulled back to allow for chest lead placement. An oxygen saturation sensor (LNOP Newborn, Masimo Corporation, Irvine, CA) was wrapped on a preductal site, usually the right wrist, before plugging this sensor into the pulse oximeter (per the manufacturer’s recommendation). The timing of placement of the devices was not simultaneous and was dependent on the providers. A stethoscope was used to listen to the HR until 1 of the devices was functioning. There was a minimum of 3 providers (an advanced life support nurse, a respiratory therapist, and a physician or neonatal nurse practitioner) at every high-risk delivery.

## Statistics

Since there are no previous randomized controlled trials of displayed and non-displayed EKG no pilot data were available for a power calculation. Therefore, a substudy was developed to consent and randomize subjects as part of the ongoing NeuPrem trial. Subjects we only exclude if they did not have a functioning EKG recording available during resuscitation (both arms). All resuscitation data were averaged over 30 seconds and the differences analyzed using two-tailed t-test. Interventions analyzed included time to first change in FiO_2_, first positive pressure ventilation, first increase in airway pressure, and first intubation time. Categorical outcomes such as demographic data (gender, antenatal steroids, intubation, and need for pressure increase were analyzed using the Chi-squared test. Statistical significance was set at less than 0.05. The data was analyzed with SPSS (IBM, ver. 22 Chicago IL).

## Results

**Fig 2:**
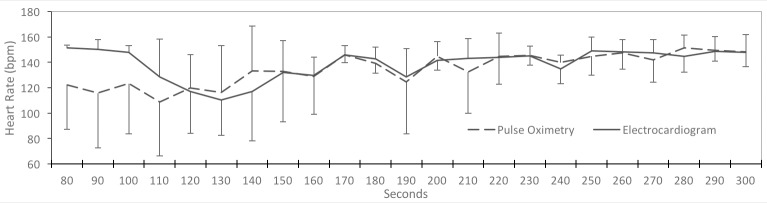
Heart rate in the first 5 minutes. Comparison of EKG and Pulse Oximeter Heart Rate in the first 5 minutes. (Beats Per Minute).

During April 2016–October 2016, 40 infants were randomized, 20 in each group. Demographics of the groups are shown in Table 1. There were no differences between the groups. Positive pressure ventilation was provided to 17% of Non-displayed vs 13% of Displayed infants (p = 0.48). Only one infant had a heart rate by EKG that was <100 at initiation of PPV (Displayed group), suggesting ineffective respirations was the primary determinant. There were no significant differences in time to clinical interventions between the groups (Table 2), despite the Displayed group having access to visible HR at 66 +/- 20 compared to 114 +/- 39 seconds for the Non-Displayed group (p < .0001). (Table 3) However in both groups the time to auscultation or palpation of the umbilical cord was less (41+/-25 seconds vs 57+/-33) in non-displayed vs displayed groups. Pulse rate was lower than EKG HR during the first 2 minutes of life, but this was not significant (Fig 2). Time to available saturation was not different between groups.

**Table 1:** Demographics.

Parameters	Heart Rate -Displayed (n = 20)	Heart Rate Displayed (n = 20)	Enrolled (n = 46)
Gestational Age	29 (2.1)	28 (2.5)	28 (2.4)
Birth Weight	1204 (468)	1185 (356)	1218 (413)
Male (%)	11 (55)	9 (45)	Male 22 (47)
C/S (%)	19 (95)	18 (90)	32 (70)
Antenatal Steroids(%)	19 (95)	19 (95)	42 (91)
Magnesium Sulfate (%)	18 (90)	18 (90)	41 (98)
1 minute Apgar (IQR)	7 (5,8)	7 (4,7)	6 (4,7)
5 minute Apgar (IQR)	8 (7,8)	8 (8,9)	8 (8,8)

**Table 2:** Delivery room interventions.

	EKG Non-Displayed (n = 20)	EKG Displayed (n = 20)	P-value
Time to Place CPAP	30 +/- 40 (n = 20)	38 +/- 6 (n = 19)	0.69
Time Positive Pressure Ventilation started	106 +/- 144 (n = 17)	77 +/- 116 (n = 16)	0.55
Number of Infants Intubated (%)	6 (30)	6 (30)	0.99
Time to Intubation	350 +/- 136	310 +/- 158	0.65
FiO_2_ Increase n, (%)	18 (90)	18 (90)	1.0 4
Time FiO_2_ Changed	148 +/- 133	106 +/- 67	0.37
Pressure Increased n (%)	5 (20)	8 (45)	0.540
Time Pressure Changed	66 +/- 62	141 +/- 184	0.48

Delivery Room Interventions (all times in seconds)

**Table 3:** Time to measured heart rate and oxygen saturation.

	EKG Non-Displayed (n = 20)	EKG Displayed (n = 20)	P-value
Time to Visible Heart Rate	114 +/- 39 (Pulse Oximeter)	66 +/- 20 (EKG)	<0.0001
Time to Heart Rate by EKG	65 +/- 19	66 +/- 20	0.97
Time to Heart Rate by Pulse Oximeter	114 +/- 39	94 +/- 38	0.11
Time to Saturation by Pulse Oximeter	86 +/- 48	90 +/- 40	0.78

Timing of Heart Rate and Saturation events (all times in seconds)

## Discussion

Obtaining an early accurate HR in neonatal resuscitation is critically important. In previous guidelines palpation of the umbilical cord or brachial pulse or auscultation were recommended as the preferred examination methods of determining HR due to their simplicity and universal availability, [12, 13] but several studies have demonstrated that clinical assessment is unreliable and inaccurate.[2],[3],[14–16] Pulse oximetry can provide an accurate HR but time to initial reading occurs much later than EKG.[9] Our study also showed that EKG provided an earlier and more accurate HR than pulse rate from an oximeter during stabilization of preterm infants allowing earlier intervention.

Iglesias et al recently compared EKG to pulse oximetry but kept the values blinded to the clinical team. [5] As in our study, they also found the EKG provided a HR reading more quickly than the pulse oximeter (87 vs 26 seconds, PO vs. EKG respectively, p < .05). They found that nearly 90% of infants that received positive pressure ventilation and close to 75% of infants who were intubated did not have a reliable pulse oximeter-derived HR measurement at the time of the intervention. Had EKG not been blinded, it would have been available in many of these cases.

We asked a different question, which was whether, if the device was available, it would make a difference in the timing of the interventions. Our study found no statistical or clinically important difference in vital signs or the time to initiate specific interventions with Displayed or Non-displayed EKG. All interventions were started earlier in the Displayed EKG group but these numbers were not significant in this small trial. This data may suggest that, in this specific environment, HR is not used as the trigger for interventions such as positive pressure ventilation, which is often initiated as a function of whether the infant is breathing, or intubation, which is undertaken for various indications in the delivery room. Other reasons could have also been that HR was still available by auscultation by the team, which may have guided interventions. While it is true that in many cases auscultation/palpation of heart rate was available first, the point of EKG/Pulse monitoring is to have a continuous representation of heart rate. While it is possible for the caregiver to auscultate or palpate the heart rate once quickly at the beginning of a resuscitation more rapidly than a monitor, this auscultation has been found to be inaccurate, and may occupy a team member who might otherwise provide critical interventions.

Another limitation of our study was the delay in EKG lead placement. In our institution, the nurse is responsible for BOTH the pulse oximetry probe and EKG lead placement. Our practice had been to place the pulse oximeter first and then the EKG leads. We believe this led to most of the infants having an available EKG/HR around 60 seconds of resuscitation. We recommend earlier EKG lead placement for future studies as well as routine clinical use.

## Conclusions

EKG provides an earlier and more accurate HR than pulse rate from an oximeter during stabilization of preterm infants at birth, allowing earlier intervention. HR may not actually drive interventions in the delivery room. Earlier EKG placement before pulse oximeter placement may affect other interventions, but this requires further research.

## Supporting information

S1 File Primary data spreadsheet. (XLSX)

S2 File Consort checklist. (DOCX)

S3 File NeuPrem protocol w/ substudy. (DOCX)
