# Supplementary material for: A pilot randomized controlled trial of EKG for neonatal resuscitation
Source: PLoS One. 2017 Nov 3;12(11):e0187730. doi: 10.1371/journal.pone.0187730 (PMC5669495; doi:10.1371/journal.pone.0187730)
Supplement: S3 File — (DOCX) [file pone.0187730.s003.docx]

# The Neu-Prem Trial: Neuromonitoring of Preterm Newborn Brain During Birth Resuscitation

**Principal Investigator**: Dr. Anup Katheria

**Co-Investigators:** Dr. Neil Finer,

**Consultant:** Dr. MJ Harbert

**Length of Study**: 2 years

# Number of subjects to be studied: 130

**Age range of subjects**: Premature newborns between 23 to 32 weeks gestation

PROPOSAL NARRATIVE.

**Goals, hypothesis and objectives.** The goal for this trial is to characterize the normal brain function of 130 very premature newborns (23 to 32 weeks gestation) during the first 72 hours of life. This is the first observational study to evaluate brain perfusion and function in 130 premature newborns ***starting at the time of birth.***

Our primary hypothesis is that simultaneously recording physiological changes of the newborn (brain activity, brain oxygenation, blood pressure, blood oxygenation, cardiac function and heart rate) beginning at birth will help us predict which babies will develop adverse problems such as bleeding in the brain (intraventricular hemorrhage, IVH) and its resultant neurological problems. We hypothesize that newborns **with low brain perfusion** and **low brain activity** at birth will have **poor outcomes** such as bleeding in the brain and death. We will measure these components of brain function using two sophisticated, non-invasive technologies. First, amplitude integrated electroencephalography (aEEG), a “simplified” EEG with four sensors (single channel), enables continuous non-invasive monitoring of cerebral activity. Second, near-infrared spectroscopy (NIRS) provided by FORE-SIGHT Elite Absolute Tissue Oximeter, is another non-invasive technology that allows continuous real-time measurement of regional tissue oxygen utilization of the brain. Both technologies are FDA approved in newborns and have been predictive of brain injury or neurodevelopmental impairment. [1-9]

Our proposal builds on an ongoing research program at a nationally recognized level 3 center with the largest number of births in California. With several completed and ongoing trials in preterm infants, our group is uniquely positioned to lead this clinical trial. (ClinicalTrials.gov:

NCT01866982, NCT02231411, NCT02195037, [10, 11]) For example, we demonstrated a heart rate can be detected using ECG leads in the delivery room much quicker compared to an oxygen saturation monitor (pulse oximeter). [12] We have also demonstrated interventions at birth (such as squeezing or milking the umbilical cord before it is cut) that provide better blood flow in babies at 6-30 hours of life. [10] It is reasonable to infer, though it is not yet proven, that these newborns *with better blood flow* had improved brain activity and improved perfusion.

**No previous study has linked measures of brain activity and oxygenation beginning at birth to neonatal outcomes.** Newborns who require extensive resuscitation (e.g. CPR or breathing assistance) are at greater risk for hypoxic-ischemic encephalopathy (HIE) and brain injury. [13, 14] Preterm infants that require more resuscitation have a higher rate of brain bleeding and death. [15]

Heart rate by ECG (electrocardiogram) is now being recommended for use in premature newborns as a method to determine heart rate compared with conventional methods of auscultation and pulse oximetry. However it is unclear as to whether the availability of a heart rate by ECG would impact the amount of resuscitation needed by premature newborns. Therefore we will make ECG available in half of the infants (by randomization at the time of delivery by the research team, in opaque sealed envelopes) and record the amount of resuscitation needed. This will allow us to implement a recommended intervention to become our standard practice. Furthermore, since heart rate is an existing outcome in the delivery room it will not change the amount of data required for parental consent.

# Methods used to achieve our goals.


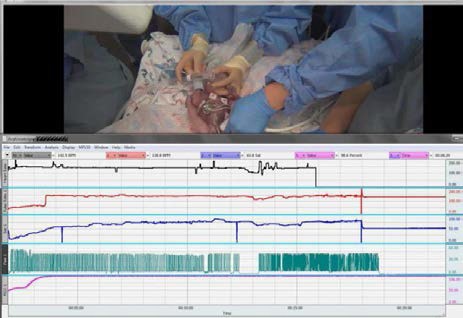
We previously demonstrated the feasibility of measuring the physiological changes of premature newborns in the delivery room (**Figure 1**). [16, 17] We routinely monitor heart rate, oxygen levels, and the amount of breathing assistance given to all of our premature babies.


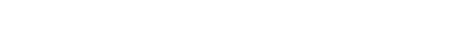


**Figure 1**

We will combine the ability to monitor brain activity using aEEG, and brain perfusion with near-infrared spectroscopy (NIRS) to capture the brain’s oxygen usage at birth. Both EEG and NIRS are acquired using inexpensive,

widely available, FDA-approved adhesive electrodes. Our last few trials have been focused on early interventions on preterm infants while monitoring the infants’ immediate

response by changes in vital signs. [11, 18, 19] These trials have provided us experience in monitoring infants *immediately upon delivery.*

Additionally, all enrolled newborns will receive a detailed brain ultrasound within 24 hours of birth and again at one week. If clinically indicated, a magnetic resonance imaging (MRI) will be performed when full-term equivalence is reached. The brain ultrasounds will alert us to any bleeding in the brain, and the MRI will reveal the full extent of any brain injury due to prematurity.

Non-invasive neural monitoring by NIRS and amplitude integrated EEG (aEEG) are able to detect differences in early brain injury.

How does NIRS work? NIRS is a technology that allows non-invasive continuous real-

time measurement of the regional tissue perfusion of organs such as the brain. Hemoglobin, which carries oxygen in the bloodstream, absorbs near-infrared light differently depending on whether the hemoglobin is carrying oxygen or not. NIRS measures the absorption of near- infrared light by the brain to measure how well the brain is using oxygen. Low brain perfusion (cerebral StO2) as measured by NIRS within the first few days of life has been shown to be associated with adverse neurological outcome and brain bleeding. [20, 21] In a single observational study, infants who had very

low cerebral StO2 in the delivery room (<10th


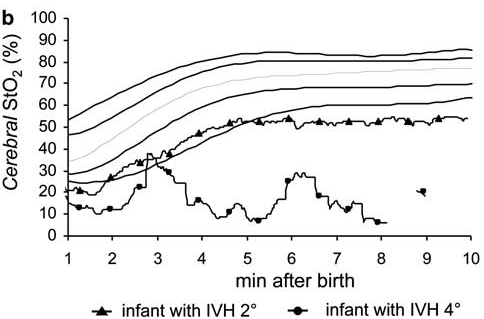


**Figure 2. Delivery room NIRS of 2 preterms that developed brain bleeds (IVH)**

percentile of normative range) were shown to have an increased risk of developing brain bleeding (**Figure 2**). [9]

How does EEG work? EEG directly

measures brain activity similar to how an

ECG measures heart activity. Recent studies

have provided data about how preterm electrical

brain activity is an indicator of brain injury. [22, 23] We will combine aEEG with NIRS to enrich

the information we obtain about the preterm brain during birth resuscitation. The interplay of

brain oxygenation and electrical brain activity has been demonstrated in a single study by a positive correlation between cerebral StO2 and aEEG in the delivery room. [24] We have started testing the application of EEG sensors in the delivery room using a simple 4-lead montage that can be applied to the forehead. [24]

Our Research Team

Dr. Anup Katheria (PI) has successfully conducted several randomized controlled trials in resuscitation and placental transfusion. [10, 11, 18, 25] He has performed studies demonstrating the benefits of UCM in premature infants, studied blood flow during caffeine and surfactant administration, and the use of continuous cardiac monitoring by electrocardiogram (ECG) in the delivery room and by Electrical Cardiometry (EC) in the intensive care unit. He successfully developed an effective collaboration with the Division of Perinatology with his previous trials. [10, 11] Dr. Neil Finer has led and designed a number of clinical trials both single site and multicenter and is considered one of the world’s experts in neonatal resuscitation. Dr. MJ Harbert is a Gerber Foundation funded neonatal neurologist who has focused on protecting the newborn brain and is currently studying the effects of resuscitation on early EEG and NIRS.

The Neonatal Research Institute Coordinators and Research Assistants, have extensive experience in multicenter as well as single center studies. They will coordinate the trial, assist in obtaining informed consents, and ensure data collection and clinical specimens are obtained in a timely fashion in keeping with accepted research standards to ensure all aspects of the protocol are followed.

# Size of the population to be studied in terms of age, gender, ethnicity, the source of subjects and the recruitment process.

In 2014 more than 9,400 babies were born at Sharp Mary Birch Hospital for Women and Newborns, and 1,500 babies were admitted into the NICU. In our most recently completed trial in premature infants with identical eligibility criteria (23-32 weeks gestation), we recruited 150 subjects in less than 2 years. (Platform Presentation: Katheria et al, E-PAS 2015). Therefore, we are confident that we will be able to enroll the 130 newborns needed over the two year grant period. This figure is also conservative to include a parental refusal rate of 20 percent since we do not anticipate many parents would refuse to participate given the minimal risks of the study. Every premature newborn from 23 to 32 weeks gestation born at Sharp Mary Birch will be considered for enrollment until we reach our target of 130 newborns over the two year period.

**Study Timeline:** We anticipate it will take 18 months to recruit 130 infants and six months to

collect and analyze data for publication and presentation at a national meeting (total 24 months). We do not anticipate that any subjects would drop out since the intervention is performed at birth.

# RECRUITMENT AND RETENTION OF SUBJECTS

***Recruitment/Consent/Stratification:*** Sharp Mary Birch Hospital for Women & Newborns and the Neonatal Research Institute (NRI) has an in-house parent advisory group that reviews trial designs to determine how parents may feel about them and whether the consent is clear and appropriate. A waiver for delayed consent is proposed in order to successfully complete the trial with minimal selection bias in a reasonable period of time. The research presents no more than minimal risk of harm to subjects. There are no known long term consequences of having leads applied to the surface of the skin. We now have compelling evidence that trials which require consent before delivery are likely to enroll a population that is not as sick as those eligible but not enrolled, which would decrease the generalizability of this large important trial. Rich et al. (Pediatrics 2010, 126: e215-e221) found that infants who were approached for participation in SUPPORT were significantly different from non-approached infants who were eligible for this trial and were born in the recruiting centers. Enrolled infants were more likely to be white, have mothers with better education and health insurance, and have prenatal care. They were less likely to be in the most immature gestational age week for this study. Antenatal Consent has been shown to limit the enrollment of the sickest infants in studies by eliminating the ability to obtain consent from

mothers with limited prenatal care and/or emergent deliveries. We believe that the use of antenatal consent would adversely affect the generalizability of the data obtained. A member of the research team will approach the parents after delivery to discuss the study. A delayed informed consent will be obtained to use the infant's data in the study and obtain neurodevelopmental follow up data.

Therefore pregnant women at <32 weeks gestation dated by their earliest ultrasound or last menstrual period will be approached for consent. In the past 12 months, the Sharp Mary Birch obstetric service had 150 premature newborns delivered by C/S. The demographics of our population are about 40 percent Caucasian, 30 percent Hispanic, 15 percent Asian Pacific

Islander, 7 percent African American and 8 percent other (Native American, etc.).

Inclusion and exclusion criteria are shown in **Table 1.**

| **Table 1** | **INCLUSION**  **Criteria** | **EXCLUSION**  **Criteria** | **REASON** |
| --- | --- | --- | --- |
| Gestational Age | 23 to 31+6 weeks |  | This GA most likely to require resuscitation interventions. Delayed consent will be obtained to allow for urgent unplanned deliveries to be included in the trial such as emergency C-Section, etc. |
| Parental refusal to participate |  | X | Parents may refuse participation antenatally or after delivery. |
| Congenital Anomalies |  | X | Increased risk for poor long-term outcomes and death. |
| Moderate to severe head bruising upon delivery |  | X | Difficulty in assessing skin integrity with dark bruising |

***Study Protocol:*** Currently, all infants taken to the resuscitation suite have recordings of heart rate, oxygenation, and airway pressure, along with simultaneous video recording. These recordings are reviewed monthly as part of our quality improvement process. Routine use of EEG or NIRS monitoring may be used per physician preference. Therefore the only additional component of the study will be the placement of electrodes (EEG) and a single sensor (NIRS) at the time of birth. ECG will be available in half of the infants per randomization during resuscitation. Upon identification of an eligible delivery, an additional research coordinator will be on standby to assist with placement of sensors.

EEG electrodes, NIRS probes, ECG electrodes and pulse oximetry will be applied within 60 seconds of the newborn being placed on the warmer. We piloted this procedure in four recent emergency C/S deliveries in which the research team successfully applied adhesive sensors within 60 seconds without interfering with resuscitation. The average time to display EEG and NIRS signals were 32 and 63 seconds, respectively. The placement of the sensors and tracing from one example are shown in **Figure 3**. (*Note – Removed for inclusion of protocol in publication*) While saturation and heart rate data will be available to the clinical team, data from both aEEG and NIRS will be blinded.

Additional monitoring will begin once the newborn is in the NICU. Electrical Cardiometry sensors (which can measure cardiac output and stroke volume) using four EC sensors (Electrical Cardiometry, Cardiotronic, La Jolla, CA) will be placed on the newborn’s chest.

Cerebral StO2, cardiac output, and stroke volume will be recorded every two seconds and linked with other variables that will be downloaded from the infant’s hospital bedside monitor.

These will include blood pressure, heart rate, and blood oxygen levels. In addition, physical exams as well as gathering the continuous bedside physiological data can be accumulated in a blinded fashion and the data will be submitted to the statistician (DG), who will remain blinded to the intervention for the duration of the study. Following study enrollment, data will be collected from the mothers and the newborns. Research data is shown in **Table 2**.

**Table 2: DATA COLLECTED FROM NEWBORNS AND MOTHERS**

**Clinical Data collected from all enrolled mothers:**

1. Age
2. Education Level
3. Insurance (private/public)
4. Primiparous (yes/no)
5. Ethnicity
6. Chorioamnionitis (yes/no)
7. Pre-Eclampsia (yes/no)
8. Duration of rupture of membranes.
9. Antenatal Steroids (yes/no)
10. Antenatal Magnesium (yes/no)
11. Cesarean Section (yes/no)
12. Reason for Cesarean Section
13. Placental Weight (grams)

**Clinical Data collected from all enrolled infants:**

1. Gestational Age
2. Gender
3. Weight (grams), length (cm) and head circumference (cm)
4. Diagnoses
5. APGARS at 1 and 5 minutes
6. Cord Gases (pH, Base deficit, arterial and venous)

**Measured Endpoints and Recorded Outcomes**

1. Amplitude Integrated EEG signal over the first 10 minutes of life
2. Cerebral StO2 by Near-InfraRed Spectroscopy within the first 10 minutes of life
3. Resuscitation (yes/no)
4. Resuscitation score (1-6)
5. DR Interventions: PPV, CPAP, intubation, chest compressions, volume, medications (yes/no)
6. Maximum inspired oxygen (FiO2) (percentage)
7. Maximum Peak Inspiratory Pressure (cm H20)
8. Time to reach heart rate 100 beats per minute (seconds)
9. Mean Blood Pressure (by non-invasive oscillimetry) at 10 minutes
10. Cardiac Output by Electrical Cardiometry (ml/kg/min) for 72 hours
11. Cerebral StO2 by Near-Infrared Spectroscopy for 72 hours
12. EEG pattern: normal- suppressed, seizure activity for 72 hours
13. Mean Arterial Blood Pressure (mm Hg)
14. Use of cardiac inotropes (dopamine, dobutamine, epinephrine) (yes/no)
15. Presence of brain injury on MRI (yes/no) (if clinically indicated)

Our engineering collaborators will help synchronize EEG and NIRS onto the existing video-analog monitoring setup to simultaneously collect information on heart rate, body oxygenation, oxygen usage by the brain, and electrical activity of the brain. We will gather their

inpatient data to look for adverse events of premature birth and resuscitation (i.e., brain bleeding, anoxic brain injury, death) and will incorporate this data with our evaluation of the neurological and physiological data gathered during the resuscitation. Additionally, all enrolled infants will have a detailed brain ultrasound within 24 hours of birth and again at one week. An MRI will be performed when they reach full-term equivalence if clinically indicated as standard of care. The brain ultrasounds will alert us to any brain bleeding, and the MRI will reveal the full extent of any brain injury of prematurity. Given the known association between extensive resuscitation with brain bleeding and death, more evidence is needed about the physiologic responses of the brain and the body during resuscitation so that resuscitation practices can be improved to promote healthy brain development and to avoid potentially devastating outcomes.

# Description of evaluation measures to assess project results and outcomes.

Our overall aim for this project is to provide preliminary data about the premature brain during the birth transition and resuscitation. Increased understanding of how the premature brain responds during this critical time will provide a physiological basis of improving the resuscitation process, will demonstrate the feasibility of such monitoring, and will be used to determine optimal practice for these vulnerable infants and improve their quality of life. After the successful conclusion of this project, we will apply specific interventions at birth in the preterm population and evaluate the resultant effects on neuro-monitoring. This will be in the context of a large randomized controlled trial powered to demonstrate improvements in long-term neurodevelopmental outcomes in children.

Given the known association between extensive resuscitation and outcomes such as bleeding in the brain and death, we expect to find physiological changes in newborns

immediately after birth and during the first 72 hours of life. We expect to find very early, potentially correctable changes in physiology that may be able to protect the brain before injury can take place.

We will also collect routine follow-up visit information at 18–30 months corrected gestational age to see if there is any correlation between NIRS and aEEG during the first 3 days of life to long term neurodevelopmental outcomes.

**STATISTICAL CONSIDERATIONS (Sample Size and Power Calculation):** All aspects of data

analysis and statistical investigations will be directed by Dr. Dale Glaser, who has formal training in Statistics and has expertise in multivariate datasets such as these.

**Statistical Analysis**: To test the primary efficacy endpoint a 2 x 2 chi-square test will be

conducted at each one minute interval, and the attendant effect size for the 2 x 2 contingency analyses (i.e., phi) will be reported. As well, relative risk (RR) and odds ratio (OR) statistics will be reported (along with 95 percent confidence intervals). For interval-level, continuous outcomes one-way ANOVA will be conducted and all assumptions will be tested/examined (normality, homogeneity of variance, etc.). Nonparametric options (e.g., Mann-Whitney) will be considered in case remedial measures such as transformations do not meet the parametric assumptions. We will compare continuous data measures averaged over each MOL using multi- variate linear regression analysis. We will use linear, logistic or generalized linear models to evaluate demographic variables and clinical outcome variables all using SPSS vs. 22.0 or Stata 11.0.

# Plan for acknowledging Foundation support for project.

The Gerber Foundation will be acknowledged in several ways. First we believe the impact of this project will have international significance and will be presented at the Neurology National Meeting, and the Pediatrics Academic Society meeting within two years of initiating this study. In addition all publications related to this trial will acknowledge the Gerber Foundation as the sponsor.

Acknowledgement for a Gerber Foundation grant of $200,000 would include: 1) Listed as a funding partner in the publication of this research trial; 2) Written press release (Gerber

Foundation approved); 3) Gerber Foundation funding will be internally communicated with all Sharp HealthCare staff via the employee intranet to over 16,000 employee; 4) Gerber Foundation will be acknowledged in the "Impact" newsletter mailed to over 9,000 Sharp

HealthCare Foundation donor constituents; 5) Gerber Foundation will be highlighted in e-News

Update, the Foundation's electronic newsletter with 1,000 subscribers; 6) Gerber Foundation’s award will be highlighted in the monthly "Philanthropy Notes" email newsletter that is delivered to approximately 16,000 employees. In addition we have been approached by the media to discuss our recent trials and any requests to present our findings would recognize the Gerber Foundation.

# Expected impact of the project nationally or regionally, potential for project replication or ways in which the project responds to the Foundation’s preference for broad impact projects.

As the first trial using multimodal monitoring for the preterm newborn brain, this data will serve as the basis for evaluating delivery room interventions potentially improving how we provide care for premature newborns at birth. In addition, by providing evidence that multimodal monitoring is predictive of outcomes in the United States, we expect to change national guidelines and practice to recommend the use of brain monitoring in the delivery room.

REFERENCES

1. Peng, S., et al., *Does Near-Infrared Spectroscopy Identify Asphyxiated Newborns at Risk of Developing Brain Injury During Hypothermia Treatment?* Am J Perinatol, 2015.
2. Massaro, A.N., et al., *Brain perfusion in encephalopathic newborns after therapeutic hypothermia.* AJNR Am J Neuroradiol, 2013. **34**(8): p. 1649-55.
3. Ancora, G., et al., *Changes in cerebral hemodynamics and amplitude integrated EEG in an asphyxiated newborn during and after cool cap treatment.* Brain Dev, 2009. **31**(6): p. 442-4.
4. Ancora, G., et al., *Early predictors of short term neurodevelopmental outcome in asphyxiated cooled infants. A combined brain amplitude integrated electroencephalography and near infrared spectroscopy study.* Brain Dev, 2013. **35**(1): p. 26-31.
5. Gucuyener, K., et al., *Use of amplitude-integrated electroencephalography (aEEG) and near infrared spectroscopy findings in neonates with asphyxia during selective head cooling.* Brain Dev, 2012. **34**(4): p. 280-6.
6. Toet, M.C. and P.M. Lemmers, *Brain monitoring in neonates.* Early Hum Dev, 2009.

**85**(2): p. 77-84.

1. Tao, J.D. and A.M. Mathur, *Using amplitude-integrated EEG in neonatal intensive care.*

J Perinatol, 2010. **30 Suppl**: p. S73-81.

1. Mathur, A.M., et al., *Utility of prolonged bedside amplitude-integrated encephalogram in encephalopathic infants.* Am J Perinatol, 2008. **25**(10): p. 611-5.
2. Fuchs, H., et al., *Brain oxygenation monitoring during neonatal resuscitation of very low birth weight infants.* J Perinatol, 2012. **32**(5): p. 356-62.
3. Katheria, A.C., et al., *The effects of umbilical cord milking on hemodynamics and neonatal outcomes in premature neonates.* J Pediatr, 2014. **164**(5): p. 1045-1050 e1.
4. Katheria, A., et al., *Umbilical cord milking improves transition in premature infants at birth.* PLoS One, 2014. **9**(4): p. e94085.
5. Katheria, A., W. Rich, and N. Finer, *Electrocardiogram provides a continuous heart rate faster than oximetry during neonatal resuscitation.* Pediatrics, 2012. **130**(5): p. e1177-81.
6. Finer, N.N., et al., *Hypoxic-ischemic encephalopathy in term neonates: perinatal factors and outcome.* J Pediatr, 1981. **98**(1): p. 112-7.
7. Miller, S.P., et al., *Patterns of brain injury in term neonatal encephalopathy.* J Pediatr, 2005. **146**(4): p. 453-60.
8. Hillemanns, P., et al., *Maternal and neonatal morbidity of emergency caesarean sections with a decision-to-delivery interval under 30 minutes: evidence from 10 years.* Arch Gynecol Obstet, 2003. **268**(3): p. 136-41.
9. Carbine, D.N., et al., *Video recording as a means of evaluating neonatal resuscitation performance.* Pediatrics, 2000. **106**(4): p. 654-8.
10. Finer, N.N., et al., *Airway obstruction during mask ventilation of very low birth weight infants during neonatal resuscitation.* Pediatrics, 2009. **123**(3): p. 865-9.
11. Katheria, A.C. and T.A. Leone, *Changes in hemodynamics after rescue surfactant administration.* J Perinatol, 2013. **33**(7): p. 525-8.
12. Katheria, A.C., et al., *A Pilot Randomized Controlled Trial of Early versus Routine Caffeine in Extremely Premature Infants.* Am J Perinatol, 2015.
13. Noori, S., et al., *Changes in cardiac function and cerebral blood flow in relation to peri/intraventricular hemorrhage in extremely preterm infants.* J Pediatr, 2014. **164**(2): p. 264-70.e1-3.
14. Zhang, Y., et al., *Cerebral near-infrared spectroscopy analysis in preterm infants with intraventricular hemorrhage.* Conf Proc IEEE Eng Med Biol Soc, 2011. **2011**: p. 1937-40.
15. Song, J., et al., *Predictive value of early amplitude-integrated electroencephalography for later diagnosed cerebral white matter damage in preterm infants.* Neuropediatrics, 2014. **45**(5): p. 314-20.
16. Benders, M.J., et al., *Early Brain Activity Relates to Subsequent Brain Growth in Premature Infants.* Cereb Cortex, 2014.
17. Pichler, G., et al., *aEEG and NIRS during transition and resuscitation after birth: promising additional tools; an observational study.* Resuscitation, 2013. **84**(7): p. 974-8.
18. Katheria, A. and T. Leone, *Altered transitional circulation in infants of diabetic mothers with strict antenatal obstetric management: a functional echocardiography study.* J Perinatol, 2012. **32**(7): p. 508-13.
